# Phase 1 study of M2698, a p70S6K/AKT dual inhibitor, in patients with advanced cancer

**DOI:** 10.1186/s13045-021-01132-z

**Published:** 2021-08-18

**Authors:** Apostolia-Maria Tsimberidou, Jamie V. Shaw, Dejan Juric, Claire Verschraegen, Amy M. Weise, John Sarantopoulos, Gilberto Lopes, John Nemunaitis, Monica Mita, Haeseong Park, Barbara Ellers-Lenz, Hui Tian, Wenyuan Xiong, Remigiusz Kaleta, Razelle Kurzrock

**Affiliations:** 1 Department of Investigational Cancer Therapeutics, The University of Texas MD Anderson Cancer Center, Unit 455, 1515 Holcombe Boulevard, Houston, TX 77030 USA; 2 EMD Serono Research and Development Institute, Inc., Billerica, MA USA; 3 Massachusetts General Hospital, Boston, MA USA; 4 Ohio State University Comprehensive Cancer Center, Columbus, OH USA; 5 Karmanos Cancer Institute, Detroit, MI USA; 6 Institute for Drug Development, Mays Cancer Center at University of Texas Health San Antonio MD Anderson Cancer Center, San Antonio, TX USA; 7 Sylvester Comprehensive Cancer Center, Miami, FL USA; 8 Gradalis, Inc., Carrollton, TX USA; 9 Samuel Oschin Comprehensive Cancer Institute, Cedars-Sinai Medical Center, Los Angeles, CA USA; 10 Washington University, St Louis, MO USA; 11 Merck KGaA, Darmstadt, Germany; 12 Merck Institute of Pharmacometrics, Merck Serono SA, Lausanne, Switzerland; 13 Moores Cancer Center, La Jolla, CA USA

**Keywords:** AKT, PI3K, p70S6K, Phase I, Advanced cancer, Clinical trial, M2698, Targeted therapy

## Abstract

**Background:**

The PI3K/AKT/mTOR (PAM) pathway is a key regulator of tumor therapy resistance. We investigated M2698, an oral p70S6K/AKT dual inhibitor, in patients with advanced cancer who failed standard therapies.

**Methods:**

M2698 was administered as monotherapy (escalation, 15–380 mg daily; food effect cohort, 240–320 mg daily) and combined with trastuzumab or tamoxifen.

**Results:**

Overall, 101 patients were treated (M2698, *n* = 62; M2698/trastuzumab, *n* = 13; M2698/tamoxifen, *n* = 26). Patients were predominantly aged < 65 years, were female, had performance status 1 and were heavily pretreated. There was a dose- and concentration-dependent inhibition of pS6 levels in peripheral blood mononuclear cells and tumor tissue. M2698 was well tolerated; the most common treatment-emergent adverse events were gastrointestinal, abnormal dreams and fatigue (serious, attributed to M2698: monotherapy, 8.1%; M2698/trastuzumab, 7.7%; M2698/tamoxifen, 11.5% of patients). The recommended phase 2 doses of M2698 were 240 mg QD (monotherapy), 160 mg QD (M2698/trastuzumab) and 160 mg QD/240 mg intermittent regimen (M2698/tamoxifen). In the monotherapy cohort, 27.4% of patients had stable disease at 12 weeks; no objective response was noted. The median progression-free survival (PFS) durations in patients with PAM pathway alterations with and without confounding markers (*KRAS*, *EGFR, AKT2*) were 1.4 months and 2.8 months, respectively. Two patients with breast cancer (M2698/trastuzumab, *n* = 1; M2698/tamoxifen, *n* = 1) had partial response; their PFS durations were 31 months and 2.7 months, respectively.

**Conclusions:**

M2698 was well tolerated. Combined with trastuzumab or tamoxifen, M2698 demonstrated antitumor activity in patients with advanced breast cancer resistant to multiple standard therapies, suggesting that it could overcome treatment resistance.

*Trial registration* ClinicalTrials.gov, NCT01971515. Registered October 23, 2013.

**Supplementary Information:**

The online version contains supplementary material available at 10.1186/s13045-021-01132-z.

## Background

The phosphatidylinositol-3-kinase (PI3K)/AKT/mammalian target of rapamycin (mTOR) (PAM) pathway is an important regulator of cell growth, proliferation, metabolism and other cellular functions [1]. PAM pathway alterations are identified in up to 30% of solid tumors [2], driving tumor growth [3]. The PAM pathway is implicated in primary and acquired resistance to anticancer therapies, including chemotherapy, endocrine therapy, tyrosine kinase inhibitors and immunotherapy, as a result of genetic alterations [4] and signaling activation [5].

PAM pathway inhibitors targeting mTOR (e.g., temsirolimus and everolimus) and PI3K (e.g., idelalisib and alpelisib) have limited efficacy owing to incomplete inhibition or reactivation of the PAM pathway, or activation of alternative prosurvival pathways [6, 7]. Inhibition of a single node in the PAM pathway (e.g., mTOR) can lead to compensatory activation, usually of AKT, via release of a negative feedback loop [7]. Compensatory prosurvival programs can be activated by PAM pathway signaling modulation or concurrent alterations in other pathways (e.g., *RAS*) that stimulate parallel tumor signaling pathways, leading to PAM pathway inhibitor resistance [8, 9]. Implementation of tumor molecular profiling and treatment with matched targeted treatments are associated with higher rates of response and progression-free and overall survival compared to non-matched therapies in patients with advanced cancer [10–13].

The use of PAM pathway inhibitors has been associated with toxicities including rash, hepatotoxicity, mucositis, hyperglycemia [14–17], and hyperlipidemia [18], which lead to treatment discontinuation [19, 20]. Thus, a PAM pathway inhibitor that does not induce the compensatory feedback loop activation and has improved tolerability would be clinically useful. M2698 is a potent, orally bioavailable, selective inhibitor of p70S6K, AKT1 and AKT3. In preclinical studies, it has demonstrated antitumor activity, the ability to inhibit proliferation of tumor cell lines harboring PAM alterations, and the capacity to cross the blood–brain barrier [21]. M2698 has the potential to block the AKT compensatory feedback loop while avoiding the adverse effects of pan-AKT inhibition, including those associated with AKT2 inhibition (e.g., hyperglycemia) [22–25].

We report the results of a phase 1 first-in-human trial of M2698 in patients with advanced metastatic cancer, refractory to standard therapies. We assessed the safety and efficacy of M2698 monotherapy and in combination with trastuzumab and tamoxifen.

## Methods

### Eligibility criteria

Eligible patients were ≥ 18 years of age with advanced metastatic cancer, whose tumors had confirmed or potentially present alterations in the PAM pathway (PAM +: e.g., PTEN, PIK3CA, AKT1, AKT3, mTOR, TSC1, or TSC2), who had exhausted all standard acceptable treatment options, with measurable disease by Response Evaluation Criteria in Solid Tumors (RECIST) criteria and tumor accessible to biopsy. Patients with asymptomatic brain metastases stable for > 4 weeks after treatment were eligible. All patients underwent cardiac function tests for eligibility (see Additional file 1). Patients with confounding *EGFR*, *KRAS* and *AKT2* alterations were excluded from monotherapy cohorts > 160 mg and both combination cohorts.

Patients eligible for the M2698/trastuzumab cohort had HER2 + refractory/recurrent metastatic breast cancer after receiving HER2-targeted therapy (trastuzumab, pertuzumab, and/or trastuzumab emtansine; PAM + not required). Patients with HER2 +, hormone-receptor-positive (HR +; i.e., estrogen- and/or progesterone-receptor-positive [ER +/PgR +]) triple-positive disease had to receive adequate hormone treatment through the study duration.

Patients eligible for the M2698/tamoxifen cohort had HR +, HER2-negative breast cancer and prior exposure to tamoxifen and/or an aromatase inhibitor with or without palbociclib (prior neoadjuvant tamoxifen was allowed if discontinued for ≥ 1 year before enrollment on the study).

Patients were enrolled in 15 centers in the USA and provided written, informed consent before any study procedures were performed. The study was conducted in accordance with ethical principles of the International Council for Harmonization guideline for Good Clinical Practice, the Declaration of Helsinki, and applicable local regulations, and was registered in www.clinicaltrials.gov (NCT01971515).

### Study design and dosing schedule

In this phase 1 study, dose escalation was followed by expansion cohorts of once-daily, oral M2698 in continuous 21-day cycles. Primary objectives were to determine the maximum tolerated dose (MTD), dose-limiting toxicities (DLTs), and recommended phase 2 dose (RP2D). Secondary objectives were to assess safety and tolerability, pharmacokinetics (PK, including food effect) and antitumor activity. Pharmacodynamic biomarker assessments were exploratory. Treatment was discontinued if there was disease progression, toxicity or consent withdrawal.

In Part 1, M2698 monotherapy was investigated in a dose escalation with a food effect cohort. In Part 2, M2698 was investigated as monotherapy (expansion cohort) to further characterize safety, tolerability and pharmacokinetics, and assess antitumor activity in PAM + patients without confounding markers (exploratory).

Two additional cohorts investigated M2698 combined with trastuzumab (M2698/trastuzumab) and tamoxifen (M2698/tamoxifen), and M2698 was escalated from 80 mg daily until the RP2D was reached. In the M2698/trastuzumab cohort, M2698 was given after trastuzumab (weekly 30–90-min intravenous infusion; doses: initial, 4 mg/kg; maintenance, 2 mg/kg). In the M2698/tamoxifen cohort, M2698 was administered concurrently with oral tamoxifen (20 mg daily) in continuous 21-day cycles except for the 240 mg dose that was given as an intermittent regimen (2 weeks on, 1 week off) (Additional file 1: Figure S1).

### Pharmacokinetics and pharmacodynamic biomarker analyses

M2698 concentration was analyzed in blood samples taken on cycle 1, days 1 and 15 at predose, 1, 2, 3, 4, 5, 6, 8, 10 and 24 h, and at predose on cycles ≥ 2, days 1, 8, and 15. Plasma PK parameters on days 1 and 15 were calculated using non-compartmental methods.

Pharmacodynamic biomarker analysis consisted of measuring levels of phospho-S6 (pS6) by flow cytometry in peripheral blood mononuclear cells (PBMCs) collected on cycle 1, days 1 and 15 at predose, 2, 4, and 8 h, and at predose on cycles ≥ 2, days 1, 8 and 15; and by immunohistochemistry in tumor biopsies obtained at baseline and on treatment (cycle 2, day 1; from the same tumor when possible). Tumor genomic analysis was performed by Foundation Medicine.

### Statistical analysis

Dose escalation analysis included all patients who experienced a DLT and those who received ≥ 80% of planned M2698 doses in cycle 1 without experiencing a DLT. Safety analysis included all patients who received ≥ 1 dose of M2698. PK analysis included all patients who received ≥ 1 dose of M2698 and had measurable plasma concentrations. Dose proportionality was tested using the power model (linear regression approach on the log -transformed scale).

Tumor response was assessed using RECIST 1.1. As for drugs with cytostatic antitumor activity, disease control rate was defined as the sum of stable disease (SD) and objective response rates; SD was considered clinically beneficial [26, 27]. Progression-free survival (PFS) was measured from treatment initiation until disease progression or death and was estimated using the Kaplan–Meier method.

## Results

### Patient demographics

From 2013 to 2018, 62 patients received M2698 monotherapy (dose escalation, *n* = 40; expansion cohort, *n* = 10; food effect cohort, *n* = 12); 13 received M2698/trastuzumab (M2698: 80 mg, *n* = 4; 160 mg, *n* = 9); and 26 received M2698/tamoxifen (M2698: 80 mg, *n* = 4; 160 mg, *n* = 9; 200 mg, *n* = 6; and 240 mg intermittent regimen, *n* = 7).

Patients were predominantly aged < 65 years, were female, had Eastern Cooperative Oncology Group (ECOG) performance status 1 and were heavily pretreated (≥ 3 prior therapies: monotherapy cohort, 50%; ≥ 5 prior therapies: M2698/trastuzumab 69.2%, M2698/tamoxifen 73.1%; Table 1). Overall, 87.1% (54/62) patients who received M2698 monotherapy were PAM + and 11.3% (7/62) were PAM + with confounding alterations.

**Table 1:** Baseline characteristics

	M2698 monotherapy (*N* = 62)	M2698/trastuzumab (*N* = 13)	M2698/tamoxifen (*N* = 26)
*Age (years)*			
< 65	45 (72.6)	9 (69.2)	18 (69.2)
65–74	12 (19.4)	3 (23.1)	4 (15.4)
75–84	5 (8.1)	1 (7.7)	4 (15.4)
Male/female	21/41 (33.9/66.1)	0/13 (0/100)	0/26 (0/100)
ECOG PS: 0/1	20/42 (32.3/67.7)	3/10 (23.1/76.9)	13/13 (50/50)
No. of prior lines of anticancer therapy: 1/2/3/4/ ≥ 5	57/47/31/21/16 (91.9/75.8/50.0/33.9/25.8)	13/12/12/11/9 (100/92.3/92.3/84.6/69.2)	25/22/23/19/19 (96.2/84.6/88.5/73.1/73.1)
*Prior therapies*			
Chemotherapy	55 (88.7)	13 (100)	22 (84.6)
Antibody therapy	6 (9.7)	3 (23.1)	3 (11.5)
Kinase inhibitor	4 (6.5)	1 (7.7)	7 (26.9)
Hormonal	10 (16.1)	5 (38.5)	19 (73.1)
Other	8 (12.9)	2 (15.4)	5 (19.2)
*Primary tumor*			
Colon	5 (8.1)	0	0
Rectum	1 (1.6)	0	0
Breast^a^	8 (12.9)	13 (100)	24 (92.3)
Pancreas	2 (3.2)	0	0
Lung	4 (6.5)	0	0
Endometrium	5 (8.1)	0	0
Salivary gland	6 (9.7)	0	0
Other	31 (50.0)	0	2 (7.7)
*Tumor molecular alterations*^*b*^			
*AKT1*	3 (4.8)	0	6 (23.1)
*AKT2*	2 (3.2)	0	0
*AKT3*	2 (3.2)	0	1 (3.8)
*PTEN*	13 (21.0)	0	3 (11.5)
*PIK3CA*	32 (51.6)	8 (61.5)	15 (57.7)
*EGFR*	3 (4.8)	0	1 (3.8)
*KRAS*	4 (6.5)	0	0
ER +	3 (4.8)	9 (69.2)	21 (80.8)
PR +	3 (4.8)	9 (69.2)	20 (76.9)
HER 1 +/2 +/3 +	1/2/0 (1.6/3.2/0)	2/3/7 (15.4/23.1/53.8)	5/3/1 (19.2/11.5/3.8)

*ECOG PS* Eastern Cooperative Oncology Group Performance Status, *N* number of patients

Data show the number of patients (percent)

^a^Patients with triple-negative breast cancer were included in the study

^b^Tumor molecular profiles were not available for all patients

### Safety

The median duration of M2698 treatment was 7.4, 6.9 and 8.4 weeks in the M2698 monotherapy, M2698/trastuzumab, and M2698/tamoxifen cohorts, respectively.

In the monotherapy dose escalation, two DLTs were observed, at 60 mg (*n* = 1; grade 3 lipase increase that resolved following treatment interruption) and 160 mg (*n* = 1; grade 3 increase in gamma-glutamyltransferase which decreased in severity following treatment interruption). The highest dose tested was 380 mg; patients with prolonged exposure to M2698 ≥ 320 mg experienced grade 2–3 gastrointestinal adverse events requiring dose reduction (monotherapy: 19.4%, M2698/trastuzumab 23.1%; M2698/tamoxifen, 15.4%). All except one of 62 patients had ≥ 1 TEAE; 34 (54.8%) patients had M2698-related TEAEs, eight (12.9%) of which were grade ≥ 3 (Table 2). The TEAEs of the monotherapy cohort by dose level are summarized in Additional file 1: Table S1. M2698-related TEAEs included gastrointestinal disorders (nausea, *n* = 12 [19.4%]; diarrhea, *n* = 7 [11.3%]), abnormal dreams (*n* = 6 [9.7%]), fatigue and tremor (*n* = 4 [6.5% each]) (Additional file 1: Table S2). One patient treated with 320 mg developed grade 3 psychosis at cycle 2, which was attributed to M2698; he was treated successfully with risperidone and M2698 was de-escalated to 200 mg. Frequently reported TEAEs are listed in Additional file 1: Table S3. Although no formal MTD was determined, the Safety Monitoring Committee determined the M2698 monotherapy RP2D as 240 mg.

**Table 2:** Overview of duration of treatment and TEAE profile across cohorts

	M2698 monotherapy (*N* = 62)	M2698/trastuzumab (*N* = 13)	M2698/tamoxifen (*N* = 26)
*Duration of M2698 therapy*			
≤ 6 weeks	26 (41.9)	6 (46.2)	10 (38.5)
> 6–12 weeks	16 (25.8)	1 (7.7)	5 (19.2)
> 12 weeks	20 (32.3)	6 (46.2)	11 (42.3)
Median, weeks (range)	7.4 (0.86–72.0)	6.9 (2.0–35.9)	8.4 (0.1–105.1)
TEAEs	61 (98.4%)	13 (100.0)	25 (96.2)
TEAEs, grade ≥ 3	36 (58.1%)	9 (69.2)	15 (57.7)
TEAE related to M2698	34 (54.8%)	10 (76.9)	22 (84.6)
TEAE related to M2698, grade ≥ 3	8 (12.9%)	3 (23.1)	5 (19.2)
TEAE l/t permanent discontinuation of any trial treatment	6 (9.7%)	3 (23.1)	9 (34.6)
TEAE l/t temporary discontinuation of M2698	29 (46.8%)	6 (46.2)	11 (42.3)
Serious TEAEs	26 (41.9)	5 (38.5)	13 (50.0)
Serious TEAE related to M2698	5 (8.1)	1 (7.7)	3 (11.5)
TEAE leading to death^a^	6 (9.7)	2 (15.4)	3 (11.5)

Data are presented as number of patients (percent) unless otherwise stated

*l/t* leading to, *TEAE* = treatment-emergent adverse event

^a^No deaths were considered to be related to M2698, trastuzumab or tamoxifen. Primary causes of death included disease progression, TEAEs not related to study drug or procedures, unknown reasons and disease related

In the M2698/trastuzumab cohort, one of 13 patients experienced a DLT (grade 3 cardiac failure) on 80 mg M2698 which resolved after treatment discontinuation; this patient was previously treated with multiple therapies, including trastuzumab, pertuzumab and lapatinib. All patients reported ≥ 1 TEAE; 10 (76.9%) patients had M2698-related TEAEs, three (23.1%) of which were grade ≥ 3 (Table 2). The most common M2698-related TEAEs were diarrhea, fatigue (*n* = 4; 30.8% each), nausea, tremor, and abnormal dreams (*n* = 1; 7.7% each) (Additional file 1: Table S2). The study was prematurely terminated due to slow enrollment and so no MTD was determined based on the doses tested.

In the M2698/tamoxifen cohort, one DLT was observed with 200 mg M2698 (grade 3 prolonged QT interval) which resolved after treatment discontinuation. All except one of the 26 patients in the cohort had ≥ 1 TEAE; 22 (84.6%) patients had M2698-related TEAEs; and five (19.2%) of these were grade ≥ 3 (Table 2). The most common M2698-related TEAEs were diarrhea (50%), nausea (50%), fatigue (15.4%) and abnormal dreams (15.4%) (Additional file 1: Table S2). The MTD was 200 mg M2698 daily, while 240 mg intermittent dosing was well tolerated.

No patient died due to a TEAE; the most common cause of death was disease progression in all cohorts. Psychiatric disorders were observed in both the M2698/trastuzumab (30.8%; 4/13) and M2698/tamoxifen (38.5%; 10/26) cohorts, were mostly mild or moderate in intensity, and were manageable with dose reduction. The most common psychiatric TEAEs were anxiety, depression and insomnia (15.4% each; *n* = 2) in the M2698/trastuzumab cohort; and anxiety (23.1%; *n* = 6), and abnormal dreams (15.4%; *n* = 4) in the M2698/tamoxifen cohort.

### Pharmacokinetics/pharmacodynamics

In the monotherapy cohort, M2698 exposure increased proportionally with dose after single and multiple administrations (Table 3, Additional file 1: Table S4, Figure S2). M2698 absorption was relatively slow (median time to maximum plasma concentration [*t*_max_], 3.08–6.28 h), the terminal half-life was long (~ 30 h) and the apparent oral clearance (CL/f) was low (~ 6 L/h). Repeated doses of M2698 resulted in a 2.12–3.38-fold accumulation on day 15, when the area under the curve for M2698 was > 30,000 ng h/mL at the highest doses of 240–380 mg (Table 3). There was no apparent food effect on the exposure of M2698 (Additional file 1: Table S4). Investigating the association between PK and safety, no clinically relevant changes in mean electrocardiogram data were observed across all cohorts; the population mean ∆QTcF at *C*_max_ on Day 15 with 380 mg (Additional file 1: Figure S3) was below the clinically significant threshold, and there was no correlation between exposure and fasting glucose levels (Additional file 1: Figure S4).

**Table 3:** Pharmacokinetic parameters of M2698 in the monotherapy and combination cohorts on day 15

Dose, mg	*n*	*C*_max_, ng/mL GeoMean (GeoCV%)	*t*_max_, hours median (range)	AUC_0–τ_, ng∙h/mL Geo mean (GeoCV%)	Accumulation ratio (*C*_max_) Geo mean (GeoCV%)
*M2698 monotherapy*					
15	3	136 (16.8)	3.00 (2.97–7.50)	2540 (6.0)	2.57 (73.6)
30	3	279 (14.5)	4.30 (1.93–6.02)	5410 (17.8)	3.19 (41.2)
60	5	566 (48.5)	5.07 (3.03–7.62)	10,000 (47.7)	3.03 (60.4)
75	4	533 (47.5)	4.05 (4.03–5.12)	9440 (39.6)	3.02 (3.2)
110	3	831 (43.3)	6.05 (3.00–6.07)	15,500 (46.8)	3.38 (55.6)
160	5	1300 (45.1)	4.08 (1.93–6.00)	23,700 (43.7)	2.40 (36.8)
200	3	1520 (100.8)	3.77 (3.50–4.05)	28,400 (91.6)	3.21 (63.5)
240	7	1920 (48.5)	4.00 (2.95–6.10)	31,300 (55.8)	2.51 (77.8)
320	6	2110 (39.6)	5.48 (3.57–6.08)	39,700 (52.3)	2.12 (67.9)
380	2	2490 (9.4)	2.50 (2.00–3.00)	41,400 (34.5)	2.20 (97.9)
*Combination with tamoxifen*					
80	2	819 (50.9)	4.5 (4.00–5.00)	16,000 (44.7)	2.31 (20.6)
160	5	1270 (48.3)	3.33 (2.95–5.58)	22,000 (42.9)	1.98 (26.6)
200	6	1380 (31.3)	3.98 (3.07–10.0)	27,000 (29.3)	3.47 (65.7)
240	4	1110 (39.9)	3.15 (2.13–4.00)	20,600 (41.7)	1.7 (40.9)
*Combination with trastuzumab*					
80	3	405 (23.7)	3.07 (2.03–5.58)	7400 (34.3)	2.00 (26.1)
160	7	2560 (52.8)	5.88 (3.05–8.08)	47,200 (49.3)	2.73 (31.5)

*AUC*_*0–τ*_ area under the plasma concentration–time curve within one dosing interval, *C*_*max*_ maximum plasma concentration, *GeoCV%* geometric coefficient of variation percent, *GeoMean* geometric mean, *t*_*max*_ time to maximum plasma concentration

**Fig. 1:**
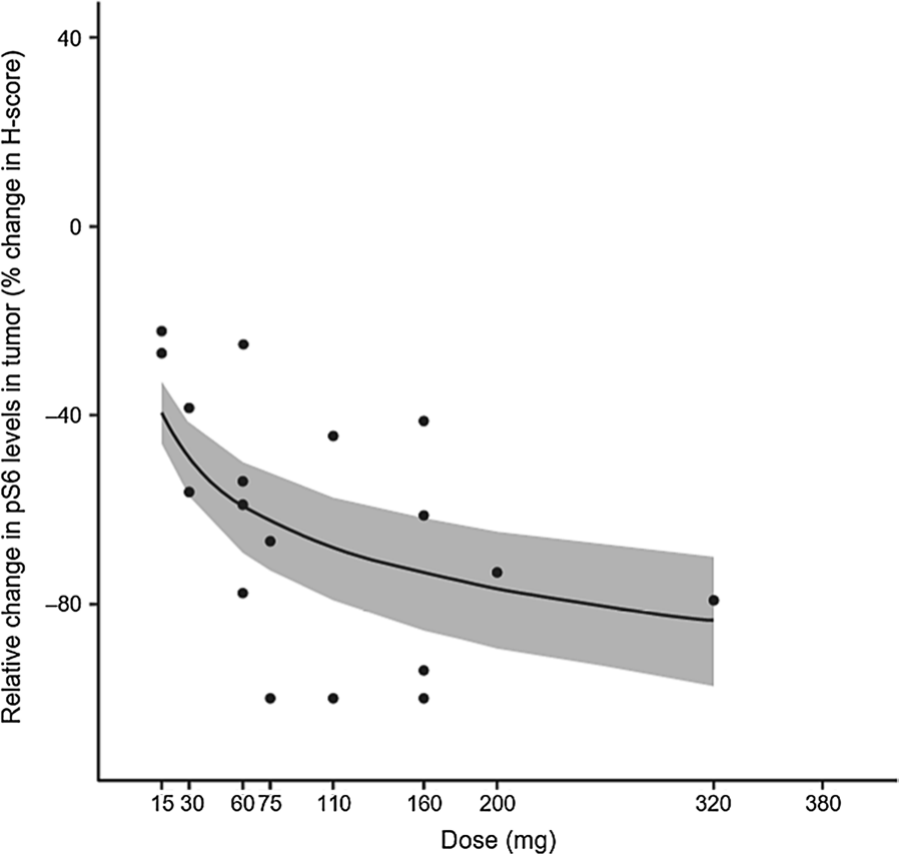
Pharmacodynamic effects of M2698 on pS6 in tumor tissue. Observed versus predicted relative change from baseline in pS6 levels in tumor tissue by dose (log-linear model). Observed data (*n* = 28) are represented by black dots and predicted data by a blue line and grey shaded area (95% confidence interval)

A monotherapy population PK model was developed, consisting of a two-compartment model with four absorption transit compartments and first-order elimination (Additional file 1: Table S5). Pharmacokinetic/pharmacodynamic modeling showed a dose- and concentration-dependent inhibition of pS6 levels in PBMCs (Additional file 1: Figure S5A) and tumor tissue (paired biopsies, 28 patients) (Fig. 1, Additional file 1: Figure S5B). A direct link of maximum drug effect (*E*_max_) model described the influence of drug concentration on pS6 levels in PBMCs over time (Additional file 1: Table S6), suggesting 50% inhibition at a plasma concentration of 1643 ng/mL. Log-linear regression and *E*_max_ models best-described the treatment effect on pS6 inhibition in tumor tissue (Additional file 1: Table S7) and suggested 70% and 80% inhibition at doses of ~ 160 and 240 mg, or AUC of 15,174 and 32,087 h.ng/mL, respectively.

### Efficacy

**Fig. 2:**
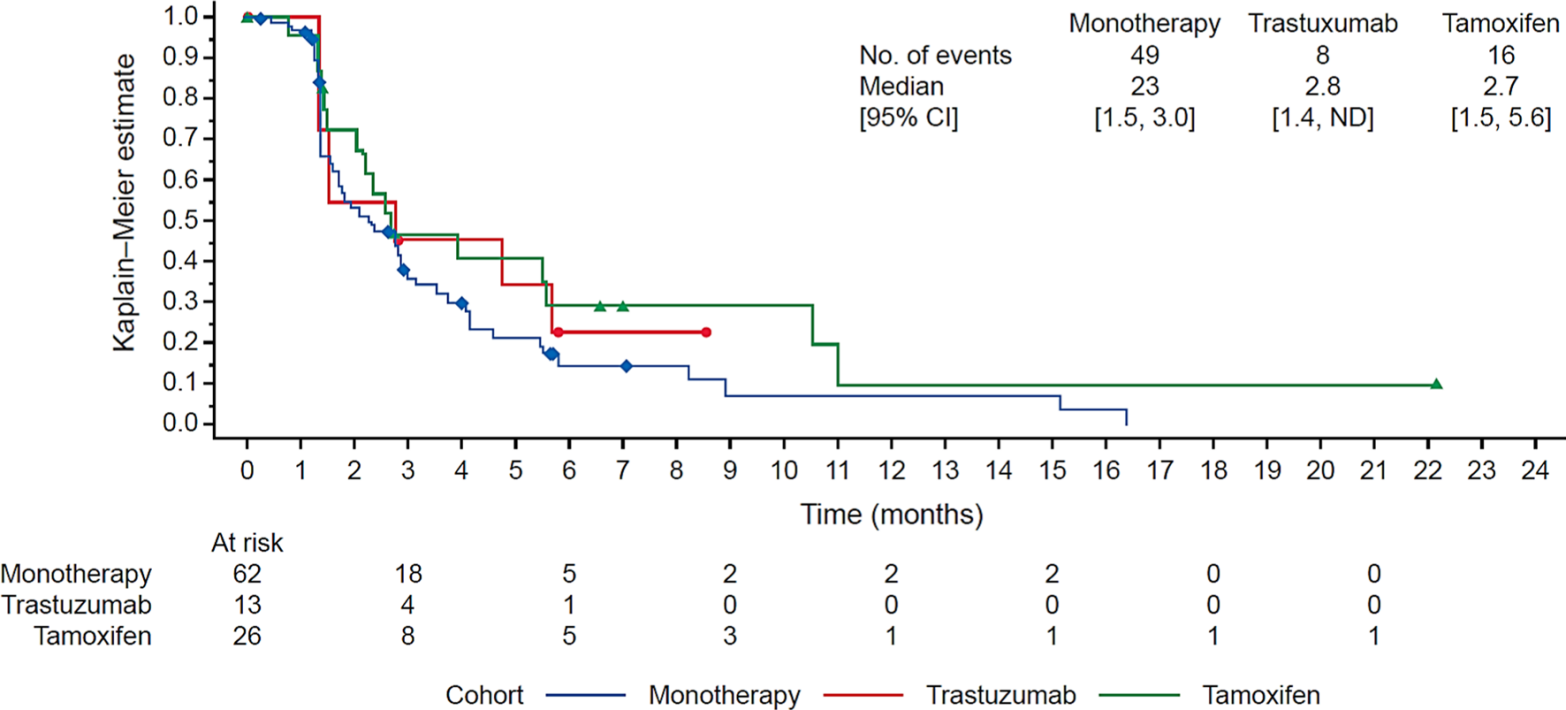
Kaplan–Meier plot of progression-free survival

In the monotherapy cohort, 25 (40.3%) patients had SD (Additional file 1: Table S8). Of the 54 PAM + patients treated with M2698 monotherapy, 22 (40.7%) had SD; no objective responses were noted. The median PFS was 2.3 months overall (Fig. 2) and 2.4 months in PAM + patients.

The presence of confounding tumor alterations appeared to impact both response and PFS. SD was observed more frequently in PAM + patients without confounding alterations (6 weeks: 21/47 [44.7%]; 12 weeks: 14/47 [29.8%]) than in PAM + patients with confounding alterations (6 weeks: 1/7 [14.3%]; 12 weeks: 1/7 [14.3%]). Additionally, the median PFS of PAM + patients without confounding alterations was 2.8 months (*n* = 47) and 1.4 months in PAM + patients with confounding alterations (*n* = 7) (Additional file 1: Table S8). Notably, all five patients with PFS ≥ 6 months (7.1–16.4 months) were PAM + without confounding alterations, and two patients remained on treatment for > 1 year.

In the combination cohorts, two patients had a partial response (PR). The first was a 66-year-old woman with triple-positive breast cancer, previously treated with four lines of therapy including letrozole, trastuzumab and lapatinib. Pretreatment tumor molecular profiling identified multiple alterations including *ERBB2* amplification and *PIK3CA* H1047R mutation. She was treated with 160 mg M2698 combined with trastuzumab and tamoxifen (20 mg daily) (per protocol for triple-positive disease). The duration of PFS was 31 months (see Additional file 1). She tolerated the treatment well except for fatigue and discontinued treatment upon disease progression.

The second patient was a 48-year-old woman with ER +, PgR +, HER2-negative breast cancer, previously treated with adjuvant cyclophosphamide and tamoxifen, taselisib and letrozole, adriamycin, and palliative radiation therapy to the lumbar spine. Pretreatment tumor molecular profiling demonstrated an *AKT1* E17K mutation. She received 240 mg M2698 combined with tamoxifen and had a PR; her PFS duration was 2.7 months (liver metastases disappeared; lung, pancreatic and bone metastases: stable; new brain metastases).

In the M2698/trastuzumab and M2698/tamoxifen cohorts, the highest clinical benefit rate and longest PFS (median PFS 3.8 and 5.5 months, respectively; *n* = 9, for each) were observed with 160 mg M2698 daily (Additional file 1: Table S9).

## Discussion

The PAM pathway is a key oncogenic mechanism and regulator of tumor resistance to existing therapies. In this phase 1 study, treatment with M2698, a dual inhibitor of p70S6K and AKT, was associated with antitumor activity in selected patients with advanced malignancies that progressed after standard-of-care therapy.

M2698 was overall well tolerated. Monotherapy dose escalation stopped at 380 mg due to the occurrence of gastrointestinal TEAEs; no MTD was reached.

Although PAM inhibitors are generally characterized by poor tolerability and narrow therapeutic index [28], commonly observed class toxicities (rash, hepatotoxicity, mucositis, hyperglycemia) [22–24] were not noted with M2698, suggesting a more favorable safety profile. Anxiety and abnormal dreams were reported and were manageable with dose reduction; one patient developed psychosis. M2698 has been shown to cross the blood–brain barrier [21], and other PAM inhibitors, such as buparlisib, have been associated with psychiatric adverse events [19]. Aberrant signaling of PI3K and AKT is implicated in the pathogenesis of some mental illnesses; therefore, pharmacological inhibition of the pathway may produce similar effects [29]. Several strategies should be implemented to mitigate the risk associated with the development of treatment-emergent psychiatric AEs. First, neuropsychological testing should be implemented in all patients prior to treatment to determine their vulnerability to develop these AEs, and during their treatment period to monitor for these events. Second, patients with preexisting psychiatric conditions should have their psychiatric medications optimized prior to initiating treatment with PAM pathway inhibitors, and they should be carefully monitored during the course of treatment. Third, novel biomarkers that predispose patients to psychiatric events should be discovered, and methods to monitor the AKT pathway activity in the brain should be developed and implemented.

Safety in combination cohorts was consistent with that of monotherapy. Dose reduction and intermittent treatment in the M2698/tamoxifen cohort were sufficient to minimize TEAEs. However, a relatively higher incidence of some TEAEs was noted in the combination cohorts, including dizziness, fatigue and gastrointestinal symptoms. Given the known toxicity profile of trastuzumab and tamoxifen, these AEs were attributed to the combination drugs.

Pharmacokinetic profiles for M2698 monotherapy and combined with tamoxifen were similar. M2698 exposure was relatively higher in combination with trastuzumab; it is unclear whether the effect was synergistic or supra-additive, and a dedicated pharmacologic study with more patients would be required to elucidate the mechanism of interaction. Preclinical pharmacodynamic and efficacy profiling demonstrated that M2698 dose-dependently inhibits pS6 in tumor tissue [21] and that > 70–80% inhibition is associated with tumor control in human breast cancer cell line-derived mouse xenograft models [30]. The clinical pharmacokinetics/pharmacodynamics model supports these findings and indicates that 70 and 80% inhibition of tumor pS6 could be achieved with doses of around 160 and 240 mg M2698, respectively. After review of all available data, including safety, clinical outcomes and the pharmacokinetic/pharmacodynamic relationship, the RP2D for M2698 was 240 mg daily as monotherapy, 160 mg daily with M2698/trastuzumab, and 160 mg daily or 240 mg intermittent regimen (2 weeks on, 1 week off) with M2698/tamoxifen.

In patients with heavily pretreated advanced cancer, the overall clinical benefit rate at 12 weeks was 27.4% with M2698 monotherapy, 38.5% with M2698/trastuzumab and 30.8% with M2698/tamoxifen. In PAM + patients treated with M2698 monotherapy, the clinical benefit rate was 40.7%, suggesting cytostatic activity of M2698, which is in line with the tumor growth inhibition observed preclinically with monotherapy treatment [21].

The two objective responses (both PRs) in the combination cohorts are noteworthy because advanced metastatic triple-positive breast cancer is associated with treatment resistance [31], and *AKT1* tumor alterations are associated with resistance to tamoxifen [32]. Therefore, there is an unmet need for effective treatments in these populations, and further investigation of these combinations is warranted.

M2698 monotherapy did not induce objective responses despite selection of tumors driven by PAM pathway defects, which may be explained by intrinsic resistance, additional molecular/compensatory pathways involved in carcinogenesis or other mechanisms. We observed that patients with PAM + tumors without confounding alterations in *KRAS*, *EGFR* and/or *AKT2* had disease stabilization with M2698 treatment, whereas PAM + patients with these markers had rapid disease progression. Alterations in *KRAS* and *EGFR* lead to activation of parallel pathways which perpetuate proliferation and prosurvival programs, which may confer resistance to PAM pathway inhibition [8, 9], while *AKT2* alterations would be M2698-specific as M2698 does not target AKT2. Future studies should endeavor to elucidate additional mechanisms of resistance to PAM inhibitors to optimize patient selection and develop effective therapies.

Numerous PAM inhibitors have been investigated in over 1150 clinical trials as of April 2021. Of these, the following molecules have been approved by the Food and Drug Administration at this time: the mTOR inhibitors everolimus [33] and temsirolimus [34]; the pan-PI3K inhibitor copanlisib [35]; the PI3Kδ inhibitors idelalisib [36] and duvelisib [37]; and the PI3Kα inhibitor alpelisib for second-line treatment of HR + metastatic breast cancer combined with fulvestrant [38, 39]. Two important characteristics may differentiate M2698 from other PAM inhibitors. First, pan-AKT and PI3K inhibitors are known to induce hyperglycemia, which is attributed to AKT2 inhibition; by sparing inhibition of AKT2, M2698 may have a more favorable and manageable safety profile. Second, the brain penetrant properties of M2698 may allow for treatment of patients with breast cancer and brain metastases, an important and currently underserved population. As this study excluded patients with known symptomatic brain lesions, this would merit further investigation in such patients.

## Conclusions

In conclusion, M2698 treatment was well tolerated as monotherapy and combined with trastuzumab or tamoxifen. The combination of M2698 may restore tumor sensitivity to endocrine therapy and to trastuzumab in patients with HR + and HER2 + breast cancer, respectively. Patients with PAM + tumors without confounding alterations appeared to benefit most from M2698 monotherapy treatment. Overall, the efficacy of M2698 monotherapy was modest, as expected based on the hypothesized cytostatic mechanism of action and advanced stage of disease in this patient population, but the observed objective responses provide encouraging results with early signs of activity and outline a potential precision oncology-based approach toward personalized treatments.

## Supplementary Information

**Additional file 1.** Supplemental methods, tables and figures.

## Data Availability

Any requests for data by qualified scientific and medical researchers for legitimate research purposes will be subject to Merck KGaA’s Data Sharing Policy. All requests should be submitted in writing to Merck KGaA’s data sharing portal (https://www.merckgroup.com/en/research/our-approach-to-research-and-development/healthcare/clinical-trials/commitment-responsible-data-sharing.html). When Merck KGaA has a co-research, co-development, or co-marketing or co-promotion agreement, or when the product has been out-licensed, the responsibility for disclosure might be dependent on the agreement between parties. Under these circumstances, Merck KGaA will endeavor to gain agreement to share data in response to requests.

## Abbreviations

DLTs: Dose-limiting toxicities
ECOG: Eastern Cooperative Oncology Group
*E*_max_: Maximum drug effect
FDA: Food and Drug Administration
MTD: Maximum tolerated dose
mTOR: Mammalian target of rapamycin
PAM: PI3K/AKT/mTOR
PBMCs: Peripheral blood mononuclear cells
PFS: Progression-free survival
PK: Pharmacokinetics
PR: Partial response
pS6: Phospho-S6
RECIST: Response Evaluation Criteria in Solid Tumors
RP2D: Recommended phase 2 dose
SD: Stable disease
TEAEs: Treatment-emergent adverse events
