# Supplementary material for: Phase 1 study of M2698, a p70S6K/AKT dual inhibitor, in patients with advanced cancer
Source: J Hematol Oncol. 2021 Aug 18;14:127. doi: 10.1186/s13045-021-01132-z (PMC8371902; doi:10.1186/s13045-021-01132-z)
Supplement: Supplementary file 1 — Additional file 1. Supplemental methods, tables and figures. [file 13045_2021_1132_MOESM1_ESM.docx]

# SUPPLEMENTAL MATERIAL

## METHODS AND RESULTS

### Eligibility criteria

Patients were excluded from the treatment if they had significantly impaired cardiac function such as unstable angina pectoris, congestive heart failure with New York Heart Association class III or IV, myocardial infarction within the last 12 months prior to trial entry; signs of pericardial effusion, serious arrhythmia (including QTc prolongation of > 450 ms for males and > 470 ms for females and/or pacemaker) or prior diagnosis of congenital long QT syndrome or left ventricular ejection fraction < 50% on screening echocardiogram. Beyond cycle 1, patients were monitored every three weeks and had to have no significant QT prolongation in order to continue treatment.

Following an interim analysis of monotherapy dose escalation cohorts ≤160 mg, the protocol was amended to exclude patients with confounding alterations of *EGFR*, *KRAS* and *AKT2*, as they were associated with unfavorable outcomes (June 2015). Subsequently, the monotherapy expansion cohort included patients with PAM+ tumors only, excluding *AKT2*-activating alterations and other genomic alterations.

In December 2015, the protocol was amended to exclude patients with any prior PAM pathway inhibitor therapy; it was revised in May 2017 to allow such treatment if it was not the immediate prior line of therapy or if it was not administered within 2 months prior to starting M2698.

### Study design and dosing schedule

DLTs were defined based on National Cancer Institute Common Terminology Criteria for Adverse Events Version 4.03 and included treatment-emergent adverse events (TEAEs) that the Safety Monitoring Committee considered of potential clinical significance such that further dose escalation would expose patients to unacceptable risk, including evidence of possible treatment-related hepatocellular injury; grade 4 neutropenia of >5 days duration or grade ≥3 febrile neutropenia; grade 4 thrombocytopenia or grade 3 thrombocytopenia with bleeding.

### Pharmacokinetics and pharmacodynamic biomarker analyses

Food effect was analyzed using an analysis of variance (performed on natural log-transformed values of area under the plasma concentration-time curve from time zero to infinity (AUC_0-∞_) and maximum plasma concentration (C_max_) including sequence, period, and fasting/fed condition as effect).

Tumor genomic sequencing analysis was performed by Foundation Medicine using the T7 solid tumor next-generation sequencing assay on formalin-fixed paraffin-embedded tissue samples and analysis by Illumina HiSeq 2500 and 4000.

### Efficacy

The first patient with objective response (66-year old woman with triple-positive breast cancer), had a PR ongoing at data cut-off, at which time she had been progression-free for 8.5 months, and was then treated off-protocol under a Single Patient Use of Investigational New Drugs. The total duration of her PFS from the time of treatment initiation was 31 months.

## SUPPLEMENTARY TABLES AND FIGURES

**Supplementary Table S1. Overview of M2698-related TEAEs by dose level – Monotherapy Cohort**

| **Primary System Organ Class Preferred Term** | **M2698**  **15 mg** | **M2698**  **30 mg** | **M2698**  **60 mg** | **M2698**  **75 mg** | **M2698**  **110 mg** | **M2698**  **160 mg** | **M2698**  **200 mg** | **M2698**  **240 mg** | **M2698**  **320 mg** | **M2698**  **380 mg** | **M2698**  **Overall** |
| --- | --- | --- | --- | --- | --- | --- | --- | --- | --- | --- | --- |
|  | n=3 | n=3 | n=6 | n=4 | n=4 | n=6 | n=3 | n=17 | n=12 | n=4 | N=62 (%) |
| **Subjects with at least one event** | 1 | 2 | 2 | 2 | 1 | 4 | 1 | 9 | 9 | 3 | 34 (54.8) |
| **Blood and lymphatic system disorders** | 0 | 0 | 0 | 0 | 0 | 1 | 1 | 0 | 1 | 0 | 3 (4.8) |
| Anaemia | 0 | 0 | 0 | 0 | 0 | 0 | 1 | 0 | 1 | 0 | 2 (3.2) |
| Lymphopenia | 0 | 0 | 0 | 0 | 0 | 1 | 0 | 0 | 0 | 0 | 1 (1.6) |
| **Cardiac disorders** | 0 | 0 | 1 | 0 | 0 | 0 | 0 | 0 | 0 | 0 | 1 (1.6) |
| Angina pectoris | 0 | 0 | 1 | 0 | 0 | 0 | 0 | 0 | 0 | 0 | 1 (1.6) |
| **Eye disorders** | 0 | 1 | 0 | 0 | 0 | 1 | 0 | 0 | 0 | 0 | 2 (3.2) |
| Dry eye | 0 | 0 | 0 | 0 | 0 | 1 | 0 | 0 | 0 | 0 | 1 (1.6) |
| Vision blurred | 0 | 1 | 0 | 0 | 0 | 0 | 0 | 0 | 0 | 0 | 1 (1.6) |
| **Gastrointestinal disorders** | 0 | 1 | 0 | 0 | 1 | 1 | 1 | 4 | 8 | 2 | 18 (29.0) |
| Constipation | 0 | 0 | 0 | 0 | 1 | 0 | 0 | 0 | 2 | 0 | 3 (4.8) |
| Diarrhoea | 0 | 1 | 0 | 0 | 0 | 0 | 0 | 2 | 4 | 0 | 7 (11.3) |
| Diarrhoea haemorrhagic | 0 | 0 | 0 | 0 | 0 | 0 | 0 | 0 | 0 | 1 | 1 (1.6) |
| Dry mouth | 0 | 0 | 0 | 0 | 0 | 1 | 0 | 0 | 0 | 0 | 1 (1.6) |
| Dyspepsia | 0 | 0 | 0 | 0 | 0 | 0 | 0 | 0 | 1 | 0 | 1 (1.6) |
| Dysphagia | 0 | 0 | 0 | 0 | 0 | 1 | 0 | 0 | 0 | 0 | 1 (1.6) |
| Gastrooesophageal reflux disease | 0 | 0 | 0 | 0 | 0 | 0 | 0 | 1 | 0 | 0 | 1 (1.6) |
| Nausea | 0 | 0 | 0 | 0 | 1 | 0 | 1 | 3 | 6 | 1 | 12 (19.4) |
| Vomiting | 0 | 0 | 0 | 0 | 0 | 0 | 0 | 1 | 0 | 1 | 2 (3.2) |
| **General disorders and administration site conditions** | 0 | 0 | 0 | 0 | 0 | 1 | 0 | 3 | 2 | 0 | 6 (9.7) |
| Energy increased | 0 | 0 | 0 | 0 | 0 | 1 | 0 | 0 | 0 | 0 | 1 (1.6) |
| Fatigue | 0 | 0 | 0 | 0 | 0 | 0 | 0 | 3 | 1 | 0 | 4 (6.5) |
| Mucosal inflammation | 0 | 0 | 0 | 0 | 0 | 0 | 0 | 0 | 1 | 0 | 1 (1.6) |
| **Investigations** | 1 | 0 | 1 | 1 | 0 | 3 | 0 | 1 | 1 | 1 | 9 (14.5) |
| Alanine aminotransferase increased | 0 | 0 | 1 | 0 | 0 | 0 | 0 | 0 | 0 | 0 | 1 (1.6) |
| Amylase increased | 0 | 0 | 1 | 0 | 0 | 0 | 0 | 0 | 0 | 0 | 1 (1.6) |
| Blood creatine phosphokinase increased | 0 | 0 | 0 | 1 | 0 | 0 | 0 | 0 | 0 | 1 | 2 (3.2) |
| Blood creatinine increased | 0 | 0 | 0 | 0 | 0 | 0 | 0 | 1 | 1 | 0 | 2 (3.2) |
| Gamma-glutamyltransferase increased | 1 | 0 | 0 | 0 | 0 | 1 | 0 | 0 | 0 | 0 | 2 (3.2) |
| Lipase increased | 0 | 0 | 1 | 0 | 0 | 0 | 0 | 0 | 0 | 0 | 1 (1.6) |
| Transaminases increased | 0 | 0 | 0 | 0 | 0 | 1 | 0 | 0 | 0 | 0 | 1 (1.6) |
| White blood cell count decreased | 0 | 0 | 0 | 0 | 0 | 1 | 0 | 0 | 0 | 0 | 1 (1.6) |
| **Metabolism and nutrition disorders** | 0 | 1 | 0 | 0 | 0 | 0 | 1 | 0 | 2 | 0 | 4 (6.5) |
| Decreased appetite | 0 | 0 | 0 | 0 | 0 | 0 | 1 | 0 | 1 | 0 | 2 (3.2) |
| Hypokalaemia | 0 | 1 | 0 | 0 | 0 | 0 | 0 | 0 | 1 | 0 | 2 (3.2) |
| Hypomagnesaemia | 0 | 0 | 0 | 0 | 0 | 0 | 0 | 0 | 1 | 0 | 1 (1.6) |
| **Nervous system disorders** | 0 | 0 | 0 | 1 | 0 | 1 | 0 | 3 | 4 | 1 | 10 (16.1) |
| Aphasia | 0 | 0 | 0 | 0 | 0 | 0 | 0 | 0 | 1 | 0 | 1 (1.6) |
| Ataxia | 0 | 0 | 0 | 0 | 0 | 0 | 0 | 0 | 1 | 0 | 1 (1.6) |
| Cognitive disorder | 0 | 0 | 0 | 0 | 0 | 0 | 0 | 1 | 0 | 0 | 1 (1.6) |
| Dizziness | 0 | 0 | 0 | 0 | 0 | 0 | 0 | 1 | 0 | 0 | 1 (1.6) |
| Dysgeusia | 0 | 0 | 0 | 1 | 0 | 1 | 0 | 0 | 0 | 0 | 2 (3.2) |
| Headache | 0 | 0 | 0 | 0 | 0 | 0 | 0 | 1 | 0 | 0 | 1 (1.6) |
| Posterior reversible encephalopathy syndrome | 0 | 0 | 0 | 0 | 0 | 0 | 0 | 0 | 0 | 1 | 1 (1.6) |
| Tremor | 0 | 0 | 0 | 0 | 0 | 0 | 0 | 1 | 3 | 0 | 4 (6.5) |
| **Psychiatric disorders** | 0 | 0 | 1 | 1 | 0 | 2 | 0 | 3 | 3 | 0 | 10 (16.1) |
| Abnormal dreams | 0 | 0 | 0 | 1 | 0 | 1 | 0 | 2 | 2 | 0 | 6 (9.7) |
| Affect lability | 0 | 0 | 1 | 0 | 0 | 0 | 0 | 0 | 0 | 0 | 1 (1.6) |
| Anxiety | 0 | 0 | 0 | 0 | 0 | 0 | 0 | 0 | 1 | 0 | 1 (1.6) |
| Delusion | 0 | 0 | 0 | 0 | 0 | 0 | 0 | 0 | 1 | 0 | 1 (1.6) |
| Depression | 0 | 0 | 0 | 0 | 0 | 0 | 0 | 1 | 1 | 0 | 2 (3.2) |
| Euphoric mood | 0 | 0 | 0 | 0 | 0 | 1 | 0 | 0 | 0 | 0 | 1 (1.6) |
| Insomnia | 0 | 0 | 0 | 0 | 0 | 1 | 0 | 0 | 1 | 0 | 2 (3.2) |
| Nightmare | 0 | 0 | 0 | 1 | 0 | 0 | 0 | 0 | 0 | 0 | 1 (1.6) |
| Paranoia | 0 | 0 | 0 | 0 | 0 | 0 | 0 | 0 | 1 | 0 | 1 (1.6) |
| **Skin and subcutaneous tissue disorders** | 0 | 1 | 1 | 1 | 0 | 2 | 0 | 0 | 3 | 0 | 8 (12.9) |
| Erythema | 0 | 0 | 0 | 0 | 0 | 1 | 0 | 0 | 0 | 0 | 1 (1.6) |
| Hyperhidrosis | 0 | 0 | 0 | 0 | 0 | 0 | 0 | 0 | 1 | 0 | 1 (1.6) |
| Onychomadesis | 0 | 0 | 1 | 0 | 0 | 0 | 0 | 0 | 0 | 0 | 1 (1.6) |
| Photosensitivity reaction | 0 | 0 | 0 | 0 | 0 | 0 | 0 | 0 | 1 | 0 | 1 (1.6) |
| Pruritus generalised | 0 | 0 | 0 | 0 | 0 | 0 | 0 | 0 | 1 | 0 | 1 (1.6) |
| Rash | 0 | 0 | 0 | 0 | 0 | 1 | 0 | 0 | 0 | 0 | 1 (1.6) |
| Rash maculo-papular | 0 | 1 | 0 | 1 | 0 | 0 | 0 | 0 | 0 | 0 | 2 (3.2) |

The monotherapy cohort includes patients from the dose escalation (n=40), food effect (n=12; 240 mg and 320 mg) and single PAM pathway mutation (n=10; 240 mg) cohorts. TEAEs are defined as events that start within the day of first dose of study treatment until 30 days after last dose of study treatment. Subjects with more than one adverse event within a System Organ Class or Preferred Term (MedDRA version 21.0) are only counted once for that System Organ Class or Preferred Term.

## Supplementary Table S2. Overview of M2698-related TEAEs (based on >5% incidence in the monotherapy cohort).

|  | **M2698 monotherapy  (N=62)** | **M2698/trastuzumab (N=13)*** | **M2698/tamoxifen (N=26)^†^** |
| --- | --- | --- | --- |
| Patients with ≥1 event | 34 (54.8) | 10 (76.9) | 22 (84.6) |
| **Gastrointestinal disorders** | | | |
| Diarrhea | 7 (11.3) | 4 (30.8) | 13 (50.0) |
| Nausea | 12 (19.4) | 1 (7.7) | 13 (50.0) |
| **General disorders and administration site conditions** | | | |
| Fatigue | 4 (6.5) | 4 (30.8) | 4 (15.4) |
| **Nervous system disorders** | | | |
| Tremor | 4 (6.5) | 1 (7.7) | 1 (3.8) |
| **Psychiatric disorders** | | | |
| Abnormal dreams | 6 (9.7) | 1 (7.7) | 4 (15.4)^§^ |

*Other M2698-related events reported in >5% (1 patient) of patients in the M2698/trastuzumab cohort (N=13): Anemia, cardiac failure, vertigo, stomatitis, vomiting, conjunctivitis, alanine aminotransferase increase, aspartate aminotransferase increase, blood creatine phosphokinase increase, ejection fraction decreased, electrocardiogram QT prolonged, decreased appetite, hypertriglyceridemia, hypocalcemia, hypomagnesemia, muscle spasms, muscular weakness, amnesia, somnolence, tremor, anxiety, depression, euphoric mood, hallucination, mania, paranoia, irregular menstruation.

^†^Other M2698-related events reported in >5% (2 patients) of patients in the M2698/tamoxifen cohort (N*=*26): anemia, constipation, dry mouth, gastroesophageal reflux disease, vomiting, asthenia, gait disturbance, platelet count decrease, weight decrease, decreased appetite, hypoalbuminemia, balance disorder, dizziness, hypoalbuminemia, pruritis, rash.

^§^Other M2698-related psychiatric events observed in a single patient (<5%) included: anxiety, hallucination (auditory), paranoia, reading disorder.

Data are number of patients (percent)

TEAE=treatment-emergent adverse event.

## Supplementary Table S3: Most commonly reported TEAEs (>10% of patients in any overall cohort)

| **TEAE, n (%)** | | **M2698  (N=62)*** | **M2698/trastuzumab (N=13)†** | **M2698/tamoxifen (N=26)‡** |
| --- | --- | --- | --- | --- |
| **Blood/lymphatic disorders** | | | | |
| Anemia | | 13 (21.0) | 2 (15.4) | 7 (26.9) |
| Neutropenia | | 1 (1.6) | 0 (0) | 3 (11.5) |
| Eye disorders, n (%) | | | | |
| Vision blurred | 3 (4.8) | | 3 (23.1) | 1 (3.8) |
| **Gastrointestinal disorders** | | | | |
| Abdominal pain | | 5 (8.1) | 0 (0) | 6 (23.1) |
| Constipation | | 14 (22.6) | 0 (0) | 9 (34.6) |
| Diarrhea | | 17 (27.4) | 6 (46.2) | 15 (57.7) |
| Dry mouth | | 3 (4.8) | 0 (0) | 4 (15.4) |
| Nausea | | 23 (37.1) | 3 (23.1) | 18 (69.2) |
| Vomiting | | 12 (19.4) | 1 (7.7) | 13 (50.0) |
| **General disorders and administration site conditions** | | | | |
| Asthenia | | 2 (3.2) | 0 (0) | 3 (11.5) |
| Chills | | 2 (3.2) | 2 (15.4) | 1 (3.8) |
| Disease progression | | 2 (3.2) | 3 (23.1) | 2 (7.7) |
| Fatigue | | 21 (33.9) | 5 (38.5) | 9 (34.6) |
| Gait disturbance | | 2 (3.2) | 0 (0) | 3 (11.5) |
| Edema peripheral | | 1 (1.6) | 1 (7.7) | 4 (15.4) |
| Pyrexia | | 11 (17.7) | 0 (0) | 2 (7.7) |
| **Infections/infestations** | | | | |
| Nasopharyngitis | 1 (1.6) | | 2 (15.4) | 1 (3.8) |
| Urinary tract infection | 5 (8.1) | | 0 (0) | 3 (11.5) |
| **Investigations** | | | | |
| ALT increased | | 4 (6.5) | 2 (15.4) | 2 (7.7) |
| AST increased | | 2 (3.2) | 2 (15.4) | 4 (15.4) |
| Blood AP increased | | 4 (6.5) | 0 (0) | 4 (15.4) |
| Blood bilirubin increased | | 2 (3.2) | 0 (0) | 3 (11.5) |
| Blood creatinine increased | | 7 (11.3) | 1 (7.7) | 1 (3.8) |
| GGT increased | | 7 (11.3) | 0 (0) | 0 (0) |
| Weight decrease | | 4 (6.5) | 0 (0) | 4 (15.4) |
| **Metabolism/nutrition disorders** | | | | |
| Decreased appetite | | 9 (14.5) | 3 (23.1) | 5 (19.2) |
| Hyperglycemia | | 1 (1.6) | 1 (7.7) | 3 (11.5) |
| Hypoalbuminemia | | 5 (8.1) | 0 (0) | 4 (15.4) |
| Hypocalcemia | | 4 (6.5) | 1 (7.7) | 3 (11.5) |
| Hypokalemia | | 4 (6.5) | 1 (7.7) | 3 (11.5) |
| Hypomagnesemia | | 2 (3.2) | 2 (15.4) | 3 (11.5) |
| Hyponatremia | | 5 (8.1) | 1 (7.7) | 3 (11.5) |
| Dehydration | | 7 (11.3) | 1 (7.7) | 2 (7.7) |
| **Nervous system disorders** | | | | |
| Dizziness | | 9 (14.5) | 0 (0) | 10 (38.5) |
| Headache | | 3 (4.8) | 4 (30.8) | 5 (19.2) |
| Tremor | | 5 (8.1) | 2 (15.4) | 1 (3.8) |
| **Musculoskeletal/connective tissue disorders** | | | | |
| Arthralgia | 1 (1.6) | | 1 (7.7) | 3 (11.5) |
| Back pain | 2 (3.2) | | 2 (15.4) | 4 (15.4) |
| Muscle spasms | 0 (0) | | 2 (15.4) | 2 (7.7) |
| Muscular weakness | 1 (1.6) | | 2 (15.4) | 1 (3.8) |
| Pain in extremity | 3 (4.8) | | 0 (0) | 3 (11.5) |
| **Psychiatric disorders** | | | | |
| Abnormal dreams | 6 (9.7) | | 1 (7.7) | 4 (15.4) |
| Anxiety | 6 (9.7) | | 2 (15.4) | 6 (23.1) |
| Depression | 3 (4.8) | | 2 (15.4) | 2 (7.7) |
| Insomnia | 2 (3.2) | | 2 (15.4) | 0 (0) |
| **Respiratory/thoracic/mediastinal disorders** | | | | |
| Cough | | 11 (17.7) | 6 (46.2) | 6 (23.1) |
| Dyspnea | | 4 (6.5) | 2 (15.4) | 2 (7.7) |
| **Skin/subcutaneous disorders** | | | | |
| Dry skin | | 0 (0) | 0 (0) | 3 (11.5) |
| Rash | | 1 (1.6) | 0 (0) | 6 (23.1) |
| **Vascular disorders** | | | | |
| Hypertension | | 4 (6.5) | 1 (7.7) | 3 (11.5) |

*Abbreviations*: ALT=alanine aminotransferase; AP=alkaline phosphatase; AST=aspartate aminotransferase; GGT=gamma glutamyl transferase.

*Grade >3 TEAEs in ≥2 patients (n, %): anemia (6; 9.7%); hyponatremia (4; 6.5%); acute kidney injury, GGT increased, urinary tract infection, pneumonia and vomiting (all 3; 4.8%); abdominal pain, acute respiratory failure, blood AP increased, blood creatine phosphokinase increased, cardiac arrest, constipation, disease progression, hypertension, nausea, pleural effusion, septic shock and small intestinal obstruction (all 2; 3.2%)

^†^Grade >3 TEAEs in ≥2 patients (n, %): disease progression (3; 23.1%); dyspnea and vision blurred (both 2; 15.4%).

^‡^Grade >3 TEAEs in ≥2 patients (n, %): hyponatremia (3; 11.5%); abdominal pain, disease progression, fatigue, hypertension (all 2; 7.7%).

Data are number of patients (percent).

## Supplementary Table S4. Pharmacokinetic parameters in the monotherapy cohort on day 1 (single dose) and in food effect cohort

| **Day 1: single dose of M2698 monotherapy** | | | | | | | |
| --- | --- | --- | --- | --- | --- | --- | --- |
| **Dose, mg** | **n** | | **C_max_, ng/mL**  **GeoMean (GeoCV%)** | | **t_max_, hours**  **Median (range)** | | **AUC_0–24_, ng∙h/mL**  **Geo mean (GeoCV%)** |
| 15 | 3 | | 53.0 (91.1) | | 3.08 (2.07–5.00) | | 759 (67.2) |
| 30 | 3 | | 87.3 (55.9) | | 4.00 (3.97–5.17) | | 1480 (52.9) |
| 60 | 6 | | 177 (38.3) | | 4.08 (2.05–5.00) | | 2730 (36.3) |
| 75 | 4 | | 269 (114.4) | | 4.83 (2.98–9.50) | | 4370 (85.2) |
| 110 | 3 | | 246 (21.7) | | 6.08 (5.98–24.3) | | 4200 (21.0) |
| 160 | 5 | | 543 (65.3) | | 4.05 (2.15–5.23) | | 8650 (49.8) |
| 200 | 3 | | 472 (110.1) | | 4.60 (4.07–5.02) | | 8180 (102.8) |
| 240 | 9 (8 for AUC) | | 590 (81.4) | | 6.25 (4.00–24.3) | | 9200 (74.5) |
| 320 | 7 (6 for AUC) | | 802 (112.0) | | 4.73 (4.00–24.1) | | 15200 (104.0) |
| 380 | 4 (2 for AUC) | | 1320 (55.0) | | 6.28 (4.12–9.52) | | 16500 (36.6) |
| **Food effect cohort*** | | | | | | | |
| **Cohort** | | **n** | | **C_max_ ,ng/mL**  **(90% CI)** | | **AUC_0-∞_ ,ng.h/mL**  **(90% CI)** | |
| Fasted | | 8 | | 769.04 (410.14–1441.99) | | 40016.18 (22818.47–70175.36) | |
| Fed | | 8 (7 for AUC) | | 934.92  (498.61–1753.02) | | 38626.33 (21989.80–67849.37) | |
| Fed/fasted ratio | | 8 (7 for AUC) | | 1.2157 (0.8058–1.8341)  p=0.3917 | | 0.9653 (0.8326–1.1191)  p=0.6504 | |

Two single doses of 240 mg M2698 were administered 7 days apart (fed then fasted states or vice versa) to assess any effect of food on pharmacokinetics; these patients then continued treatment with either 240 mg/day or 320 mg/day M2698 as part of the dose expansion cohort. (240 mg, n=7; 320 mg, n=5)

^*^Fed vs fasting following single dose of 240 mg M2698. Main analysis performed on natural log-transformed values of AUC_0-∞_ and C_max_ including sequence, period and fasting/fed condition as effect. For one of the eight evaluable patients, no AUC under fed conditions was determined. The difference (fed–fasted) in least square means and 90% CI from the model have been back transformed with the exponential function to be displayed on the original scale.

AUC_0–24_=area under the plasma concentration–time curve from 0–24 hours; AUC0–τ=area under the plasma concentration–time curve within 1 dosing interval; CI=confidence interval; C_max_=maximum plasma concentration; GeoCV%=geometric coefficient of variation percent; GeoMean=geometric mean; t_max_=time to maximum plasma concentration.

## Supplementary Table S5. Population pharmacokinetic model parameter estimates

| **Parameter** | **Estimate** | **%RSE** | **95% CI** | **IIV(+%RSE)** | **Shrinkage** |
| --- | --- | --- | --- | --- | --- |
| CL^1^ (L/h) | 7.06 | 7.0 | 6.09/8.03 | 0.91(62.0) | 19.9% |
| Vc^2^ (L) | 292 | 13.5 | 215/370 | 1.42(43.4) | 13.2% |
| F1 | 1.00 | FIXED |  | 0.91(67.2) | 22.0% |
| Vp (L) | 53.0 | 46.9 | 4.33/102 |  |  |
| KA (h^-1^) | 2.02 | 11.6 | 1.56/2.48 | 0.73(71.9) | 1.0% |
| Q (L/h) | 18.0 | 52.6 | -0.544/36.6 |  |  |
| Additive RSV | 3.44 | 87.7 | -2.47/9.35 |  |  |
| Proportional RSV | 0.163 | 9.9 | 0.132/0.195 |  |  |
| CLT | -0.000315 | 29.2 | -0.000495/-0.000135 | 4.3E-04(32.6) | 16.2% |
| ALB on Vc | 0.0247 | 44.2 | 0.00329/0.0461 |  |  |
| BSA on Vc | 1.17 | 29.3 | 0.499/1.84 |  |  |

^1^Full equation for CL=exp((log(7.06) + ETA_CL) + (-0.000315 + ETA_CLT)*Time)
^2^Full equation for Vc=292 exp(0.0247 *(ALB-40.51)) *( 1 + 1.17*(BSA - 1.79)) * exp(ETA_Vc)

*Abbreviations*: ALB=albumin; BSA=body surface area: CI=confidence interval; CL=clearance; CLT= time-dependent parameter on CL; ETA=deviation; F1=bioavailability; IIV=inter-individual variability; PBMC = peripheral blood mononuclear cell; KA=absorption rate constant; Q=flow rate; RSE=relative standard error; RSV=residual variability; Vc= Volume of distribution of the central compartment; Vp=Volume of distribution of the peripheral compartment.

## Supplementary Table S6. NONMEM parameter estimates for pharmacokinetic/pharmacodynamic model of pS6 in PBMCs

| **Parameter** | **Estimate** | **%RSE** | **95% CI** | **IIV** | **Shrinkage** |
| --- | --- | --- | --- | --- | --- |
| E_max_ | 0.148 | 17.4% | 0.0978/0.198 | 0.166 | 71.3% |
| Baseline | 9.12 | 1.1% | 8.93/9.31 | 0.581 | 1.0% |
| EC50 (ng/mL) | 1643 | 28.3% | 732/2554 |  |  |

*Abbreviations*: CI=confidence interval; EC50= concentration to give the half-maximal effect; E_max_= maximum drug effect; PBMC = peripheral blood mononuclear cell; RSE=relative standard error.

## Supplementary Table S7. Parameter estimates for pharmacokinetic/ pharmacodynamic models of pS6 in tumor tissue using dose and area under the curve (AUCT, calculated as dose divided by apparent clearance at time of tissue biopsy) as exposure metrics

log-linear regression model of dose – tumor pS6: y ~ slope *log(Dose)

| **Parameter** | **Estimate (SE)** |
| --- | --- |
| slope | -14.50 (1.12) |

E_max_ regression model of AUC – tumor pS6: y ~ (E_max_ *AUC) / (EC_50_ + AUC)

| **Parameter** | **Estimate (SE)** |
| --- | --- |
| E_max_ | -91.73 (13.50) |
| EC50 | 4706.9 (2489.7) |

*Abbreviations*: AUC=area under the curve; EC50= concentration to give the half-maximal effect; E_max_= maximum drug effect; SE=standard error.

## Supplementary Table S8. Progression-free survival and response in patients receiving M2698 monotherapy

| Dose level, mg | 15 | 30 | 60 | 75 | 110 | 160 | 200 | 240 | 320 | 380 | Total |
| --- | --- | --- | --- | --- | --- | --- | --- | --- | --- | --- | --- |
| **All patients** | | | | | | | | | | | |
| No. of patients | N=3 | N=3 | N=6 | N=4 | N=4 | N=6 | N=3 | N=17 | N=12 | N=4 | N=62 |
| Response, n (%)  SD at wk 6  PD  NE | 2 (66.7) 1 (33.3) 0 (0.0) | 3 (100) 0 (0.0) 0 (0.0) | 1 (16.7) 4 (66.7) 1 (16.7) | 1 (25.0) 3 (75.0) 0 (0.0) | 1 (25.0) 2 (50.0) 1 (25.0) | 2 (33.3) 4 (66.7) 0 (0.0) | 2 (66.7) 1 (33.3) 0 (0.0) | 6 (35.3) 8 (47.1) 3 (17.6) | 5 (41.7) 5 (41.7) 2 (16.7) | 2 (50.0) 0 (0.0) 2 (50.0) | 25 (40.3) 28 (45.2) 9 (14.5) |
| SD at wk 12, n (%) | 2 (66.7) | 3 (100.0) | 1 (16.7) | 0 (0.0) | 1 (25.0) | 2 (33.3) | 2 (66.7) | 3 (17.6) | 2 (16.7) | 1 (25.0) | 17 (27.4) |
| Median PFS, months | 4.1 | 4.1 | 1.4 | 1.4 | 1.3 | 1.6 | 4.1 | 2.0 | 2.8 | 6.2 | 2.3 |
| **PAM+ patients** | | | | | | | | | | | |
| No. of patients | N=3 | N=2 | N=4 | N=2 | N=3 | N=6 | N=3 | N=16 | N=11 | N=4 | N=54 |
| Response, n (%)  SD at wk 6  PD  NE | 2 (66.7) 1 (33.3) 0 (0.0) | 2 (100) 0 (0.0) 0 (0.0) | 0 (0.0) 3 (75.0) 1 (25.0) | 1 (50.0) 1 (50.0) 0 (0.0) | 1 (33.3) 1 (33.3) 1 (33.3) | 2 (33.3) 4 (66.7) 0 (0.0) | 2 (66.7)  1 (33.3) 0 (0.0) | 5 (31.3) 8 (50.0)  3 (18.8) | 5 (45.5) 5 (45.5) 1 (9.1) | 2 (50.0) 0 (0.0) 2 (50.0) | 22 (40.7) 24 (44.4) 8 (14.8) |
| SD at wk 12, n (%) | 2 (66.7) | 2 (100.0) | 0 (0.0) | 0 (0.0) | 1 (33.3) | 2 (33.3) | 2 (66.7) | 3 (18.8) | 2 (18.2) | 1 (25.0) | 15 (27.8) |
| Median PFS, months | 4.1 | 10.3 | 1.4 | 2.1 | 8.2 | 1.6 | 4.1 | 1.9 | 2.8 | 6.2 | 2.4 |
| **PAM+ patients with confounding markers** | | | | | | | | | | | |
| No. of patients | N=1 | N=0 | N=2 | N=1 | N=1 | N=2 | N=0 | N=0 | N=0 | N=0 | N=7 |
| Response, n (%)  SD  PD  NE | 1 (100) 0 (0.0) 0 (0.0) | – | 0 (0.0) 1 (50.0) 1 (50.0) | 0 (0.0) 1 (100) 0 (0.0) | 0 (0.0) 1 (100) 0 (0.0) | 0 (0.0) 2(100.0) 0 (0.0) | – | – | – | – | 1 (14.3) 5 (71.4) 1 (14.3) |
| SD at wk 12, n (%) | 1 (100) | – | 0 (0.0) | 0 (0.0) | 0 (0.0) | 0 (0.0) | – | – | – | – | 1 (14.3) |
| Median PFS, months | ND | – | 1.4 | 1.4 | 1.3 | 1.4 | – | – | – | – | 1.4 |
| **PAM+ patients without confounding markers** | | | | | | | | | | | |
| No. of patients | N=2 | N=2 | N=2 | N=1 | N=2 | N=4 | N=3 | N=16 | N=11 | N=4 | N=47 |
| Response, n (%)  SD at wk 6  PD  NE | 1 (50.0) 1 (50.0) 0 (0.0) | 2 (100.0) 0 (0.0) 0 (0.0) | 0 (0.0) 2(100.0) 0 (0.0) | 1(100.0) 0 (0.0) 0 (0.0) | 1 (50.0) 0 (0.0) 1 (50.0) | 2 (50.0)  2 (50.0) 0 (0.0) | 2 (66.7) 1 (33.3)  0 (0.0) | 5 (31.3) 8 (50.0) 3 (18.8) | 5 (45.5) 5 (45.5) 1 (9.1) | 2 (50.0) 0 (0.0) 2 (50.0) | 21 (44.7) 19 (40.4) 7 (14.9) |
| SD at wk 12, n (%) | 1 (50.0) | 2 (100.0) | 0 (0.0) | 0 (0.0) | 1 (50.0) | 2 (50.0) | 2 (66.7) | 3 (18.8) | 2 (18.2) | 1 (25.0) | 14 (29.8) |
| Median PFS, months | 4.1 | 10.3 | 1.4 | 2.8 | 15.1 | ND | 4.1 | 1.9 | 2.8 | 6.2 | 2.8 |

*Abbreviations*: ND = not determined; NE = non-evaluable; No. = number; PAM+ = PI3K/AKT/mTOR-positive; PD = progressive disease; PFS = progression-free survival; SD = stable disease; wk = week.

## Supplementary Table S9. Progression-free survival and response in patients receiving M2698 in combination with trastuzumab or tamoxifen

|  | **M2698/trastuzumab** | | | **M2698/tamoxifen** | | | | |
| --- | --- | --- | --- | --- | --- | --- | --- | --- |
| Dose level, mg | 80 | 160 | Overall | 80 | 160 | 200 | 240 | Total |
| No. of patients | N=4 | N=9 | N=13 | N=4 | N=9 | N=6 | N=7 | N=26 |
| Response, n (%)  PR  SD  PD  NE | 0 (0.0) 1 (25.0) 2 (50.0) 1 (25.0) | 1 (11.1) 4 (44.4)  2 (22.2) 2 (22.2) | 1 (7.7) 5 (38.5) 4 (30.8) 3 (23.1) | 0 (0.0) 2 (50.0) 0 (0.0) 2 (50.0) | 0 (0.0) 6 (66.7) 2 (22.2) 1 (11.1) | 0 (0.0) 2 (33.3) 3 (50.0) 1 (16.7) | 1 (14.3) 3 (42.9) 1 (14.3) 2 (28.6) | 1 (3.8) 13 (50.0) 6 (23.1) 6 (23.1) |
| PFS rate, n (%) at 6 months | ND | 16.7 (0.9, 50.8) | 22.7 (3.8, 51.1) | 0.0 | 41.5 (6.9, 74.8) | 40.0 (5.2, 75.3) | 20.0 (0.8, 58.2) | 29.0 (11.0, 50.0) |
| PFS rate*, n (%) at 12 months | ND | ND | ND | 0.0 | 0.0 | 20.0 (0.8, 58.2) | ND | 9.7 (0.7, 33.2) |
| PFS, months  Median, 95% CI | 1.4 (1.4, ND) | 3.8 (1.3, ND) | 2.8 (1.4, ND) | 2.2 (2.1, 5.6) | 5.5 (0.8, 11.0) | 1.5 (1.3, ND) | 2.7 (2.3, ND) | 2.7 (1.5, 5.6) |
| PFS events, n (%) Death PD | 0 (0.0) 2 (5.0) | 1 (11.1) 5 (55.6) | 1 (7.7) 7 (53.8) | 1 (25.0) 2 (50.0) | 1 (11.1) 4 (44.4) | 0 (0.0) 4 (66.7) | 1 (14.3) 3 (42.9) | 3 (11.5) 13 (50.0) |
| Disease control rate at wk 6, n (%) | 1 (25.0) | 5 (55.6) | 6 (46.2) | 2 (50.0) | 6 (66.7) | 2 (33.3) | 4 (57.1) | 14 (53.8) |
| Clinical benefit rate at wk 12, n (%) | 1 (25.0) | 4 (44.4) | 5 (38.5) | 1 (25.0) | 4 (44.4) | 2 (33.3) | 1 (14.3) | 8 (30.8) |

*Abbreviations*: ND = not determined; NE = non-evaluable; No. = number; PD = progressive disease; PFS = progression-free survival; PR=partial response; SD = stable disease; wk = week.

## Supplementary Figure S1. Study Schema


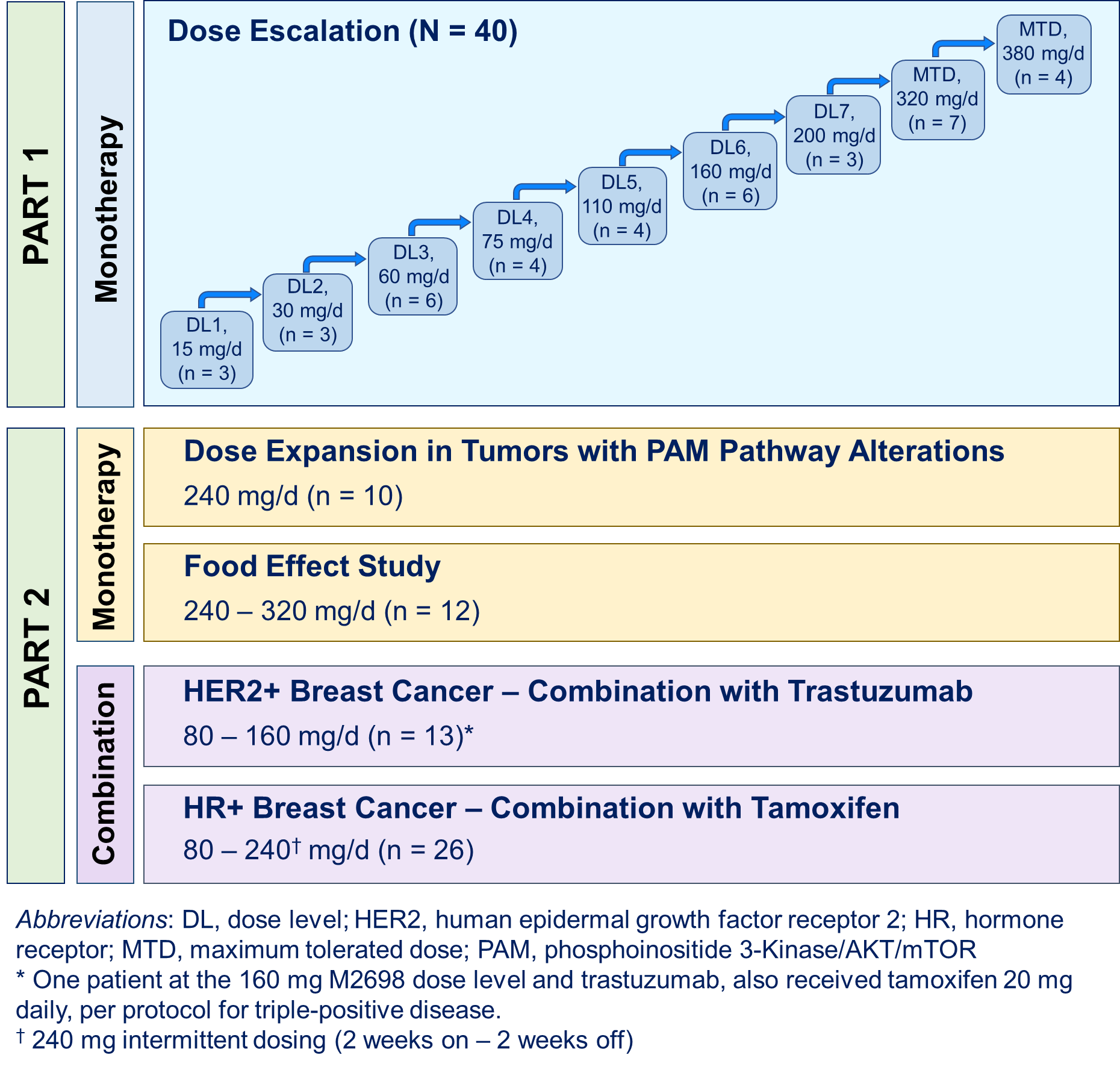


## Supplementary Figure S2. Plasma concentration vs time after a single dose of M2698 under fasting and fed conditions (semi-log scale).


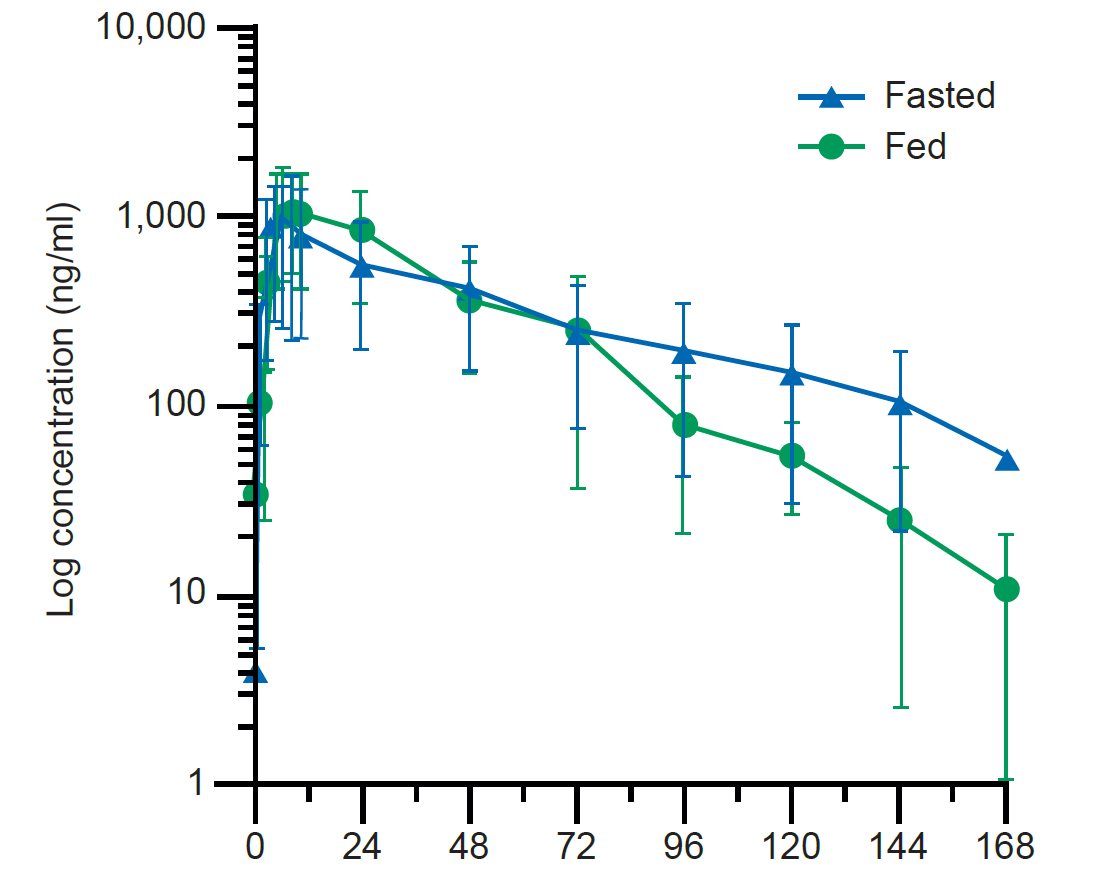


Data are presented as mean ± standard deviation.

## Supplementary Figure S3. Plot of modelled change in QT interval (Fridericia's correction) vs M2698 concentration


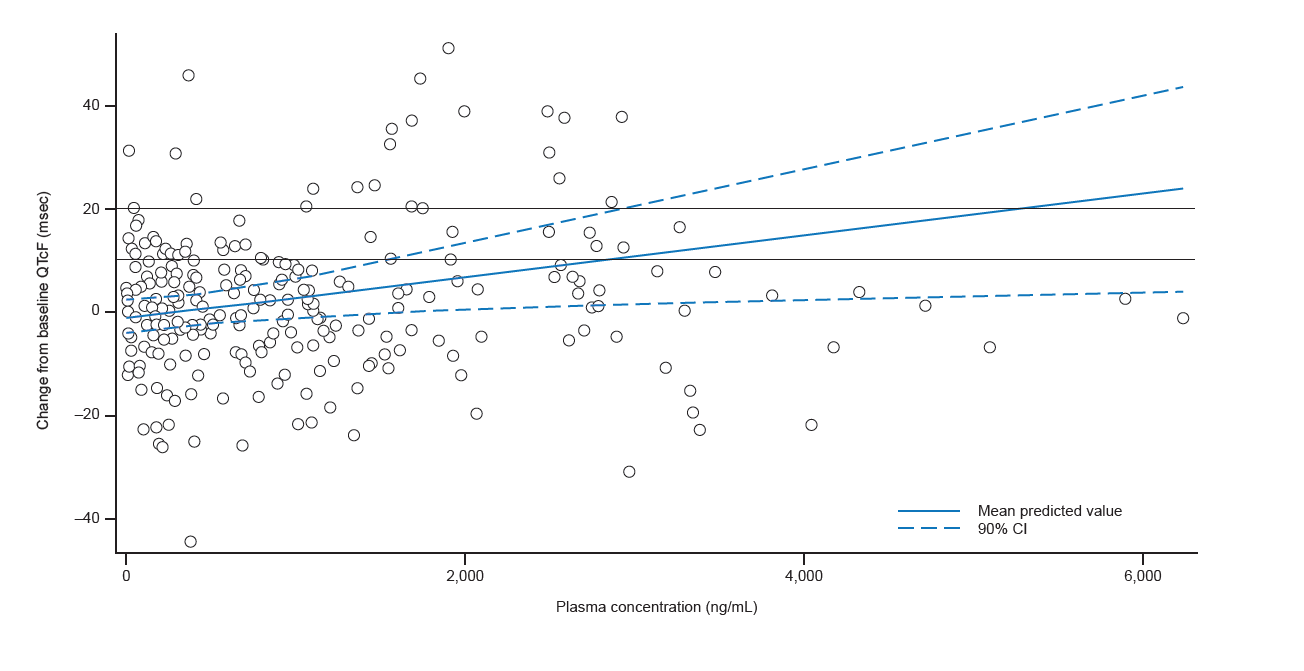


Data from the M2698/tamoxifen cohort are not included.

## Supplementary Figure S4. Change from baseline in fasting glucose levels vs M2698 plasma concentration


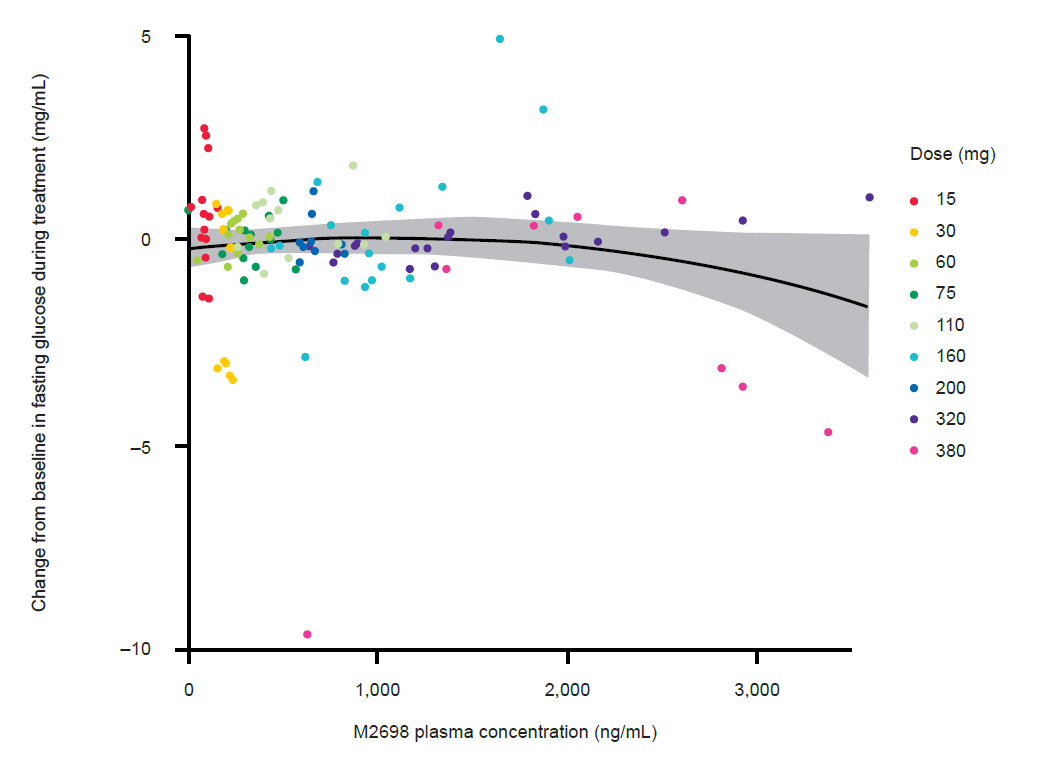


## Supplementary Figure S5. Pharmacodynamic effects of M2698 on pS6 in PBMCs and tumor tissue.

**A**) Change from baseline in pS6 levels in PBMC vs observed M2698 plasma concentration; **B**) Observed vs predicted relative change from baseline in pS6 levels in tumor tissue by concentration (E_max_ model); observed data are represented by black dots and predicted data by a blue line and grey shaded area (95% confidence interval).

**A**


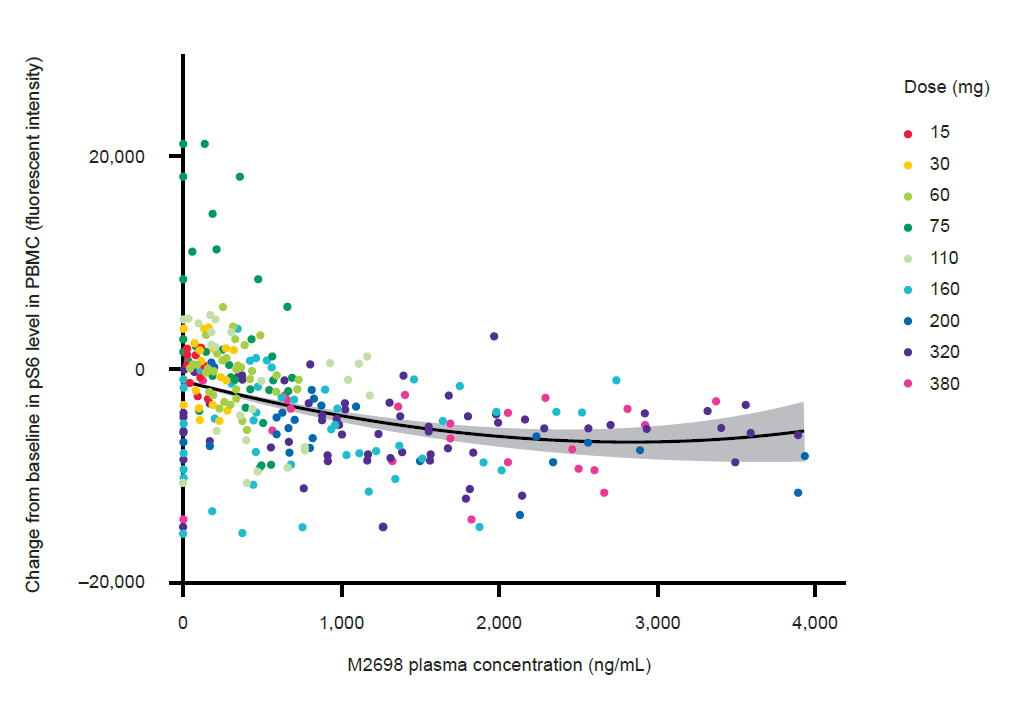


**B**


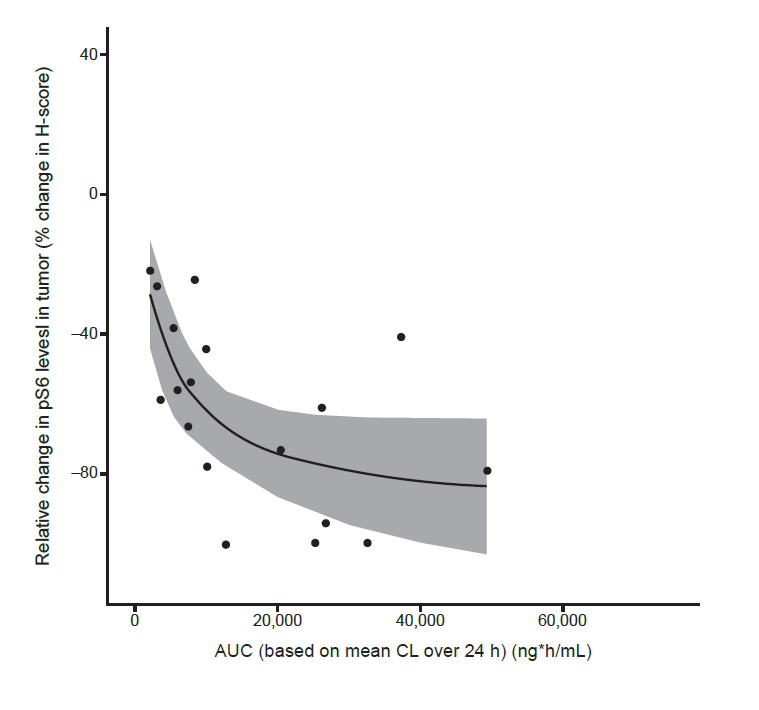


## 
